# Insight into the role of pre-assembly and desolvation in crystal nucleation: a case of *p*-nitro­benzoic acid

**DOI:** 10.1107/S2052520619009478

**Published:** 2019-09-18

**Authors:** Shuyi Zong, Jingkang Wang, Hao Wu, Qi Liu, Yunhui Hao, Xin Huang, Dehui Wu, Guanchen Zhou, Hongxun Hao

**Affiliations:** a School of Chemical Engineering and Technology, Tianjin University, Tianjin, 300072, People’s Republic of China; b Collaborative Innovation Center of Chemical Science and Engineering, Tianjin, 300072, People’s Republic of China; c China Nuclear Power Engineering Co. Ltd, Beijing Institute of Nulcear Chemistry Engineering, Beijing, 100840, People’s Republic of China

**Keywords:** crystal nucleation, solution chemistry, solute–solvent interaction, desolvation, nucleation dynamics

## Abstract

As one of the most important phenomena in crystallization, the crystal nucleation process has always been the focus of research. In this work, influences of pre-assembly species and the desolvation process on the crystal nucleation process were studied. *p*-Nitro­benzoic acid (PNBA) was taken as a model compound to investigate the relationship between solution chemistry and nucleation kinetics in seven different solvents. One unsolvated form and four solvates of PNBA were obtained and one of the solvates was newly discovered. The nucleation behaviours and nucleation kinetics of PNBA in the seven solvents were studied and analyzed. Density functional theory (DFT) and solvation energy calculation were adopted to evaluate the strength of solute–solvent interactions. Vibrational spectroscopy combined with molecular simulation was applied to reveal the pre-assembly species in the solution. Based on these results, a comprehensive understanding of the relationship between molecular structure, crystal structure, solution chemistry and nucleation dynamics was proposed and discussed. It was found that the structural similarity between solution chemistry and crystal structure, the interaction between specific sites and the overall strength of solvation will jointly affect the nucleation process.

## Abstract 2

The pathway and hybrid control mechanism of crystal nucleation were studied through both experimental and computational investigation on the role of pre-assembly and desolvation in the nucleation process.

## 1. Introduction   

Nucleation is a key step in the crystallization process which has decisive influence on the crystal-size distribution and polymorphism of the final products. Unfortunately, although crystal nucleation from solution is common, there is insufficent understanding of the early stage in the crystal formation process and the nucleation mechanism is still not fully understood from the molecular level. In recent years, solution chemistry has turned out to be a useful tool to explore the molecular assembly path in the nucleation process due to the significant influence of solute–solvent interactions on the molecular self-assembly process (Du *et al.*, 2015 ▸; Gebauer *et al.*, 2014 ▸; Davey *et al.*, 2013 ▸). Besides, the rapid development of computational techniques and the notable improvement of analysis techniques have made it possible to study larger molecular clusters more efficiently. Plenty of work therefore has been carried out to interpret the evolution of the so-called ‘growth units’ in the crystallization nucleation process (Gavezzotti *et al.*, 1997 ▸; Chen & Trout, 2008 ▸; Tommaso, 2013 ▸; Zeglinski *et al.*, 2018 ▸). The structural correlation between solution aggregates and crystal syntheses has been investigated by studying the structural evolution process from solute molecules to supramolecular syntheses (Parveen *et al.*, 2005 ▸; Byrn *et al.*, 1976 ▸; Bernstein & Hagler, 1978 ▸; Habgood, 2012 ▸).

It has been known that crystal nucleation is very sensitive to solution chemistry in the aspects of nucleation rate and nucleation polymorphism (Ostwald, 1897 ▸; Teychené & Biscans, 2008 ▸). In recent years, many studies have shown that the structural arrangement of solute molecular clusters in solution will affect subsequent nucleation, and the synthons formed in solution will facilitate the nucleation and formation of the corresponding crystal structure (Cruz-Cabeza *et al.*, 2017 ▸; Sullivan *et al.*, 2014 ▸). The relationship between solution chemistry and nucleation was first discovered from 2,6-di­hydroxy­benzoic acid (Davey *et al.*, 2001 ▸), and the direct evidence was first reported for tetrolic acid (Parveen *et al.*, 2005 ▸). Subsequent spectroscopy studies supported the idea that the transformation from molecular self-assemblies to crystalline growth units was significantly affected by the solute–solvent interactions during the first stage of crystallization (clustering and nucleation), and these interactions could lead to the formation of supramolecular syntheses which could indirectly reflect the presence of synthons in crystalline structures (Hunter *et al.*, 2012 ▸; Davey *et al.*, 2006 ▸; Kulkarni *et al.*, 2012 ▸; Mattei *et al.*, 2013 ▸). The neutron scattering method was applied to carry out the structural analysis in detail. The results revealed the effects of solvents on the supersaturated state and confirmed the importance of solute desolvation in forming molecular clusters which would be further developed into nuclei and crystals (Burton *et al.*, 2010 ▸).

In order to interpret these experimental phenomena and simulate crystal nucleation and growth processes, many efforts have been devoted to simulation techniques. Chen & Trout (2008 ▸) used molecular dynamic simulation to analyze the influence of solvent on the solute synthon formation and concluded that the solute–solvent interactions played an important role in the self-assembly process of solute in solution. Khamar *et al.* (2014) ▸ have revealed the direct relationship between the strength of solute–solvent interaction and the experimental nucleation rates of salicylic acid via a modelling approach. The results indicated that the relative difficulty of nucleation was related to the strength of solvation and the associated difficulty of forming hydrogen-bonded dimers (Khamar *et al.*, 2014 ▸; Yang *et al.*, 2014 ▸). However, in most investigated systems, consistent molecular conformations in solution state and crystal state were due to the weak solvation effect. It is important to take strong solvation into account when exploring the nucleation transition states both experimentally and computationally.

In order to better understand the relationship between the molecular structure, crystal structure, solution chemistry and nucleation kinetics, investigations on the relationship between solution chemistry and nucleation kinetics were carried out using *p*-nitro­benzoic acid (PNBA) as the model compound. The nucleation process of PNBA in seven solvents [chloro­form, aceto­nitrile, methanol, di­methyl sulfoxide (DMSO), *N*,*N*-di­methyl­formamide (DMF), *N*-methyl pyrrolidone (NMP), *N*,*N*-di­methyl­acetamide (DMA)] was investigated through a combination of spectroscopic techniques (NMR, FTIR) and computational methods [including density functional theory (DFT), molecular dynamics and free-energy techniques]. The crystallization products (both pure crystal and solvates) and the self-association properties of PNBA in these seven solvents were studied in detail and the crystal nucleation rates were determined based on the induction time measurements at different supersaturations. The influences of solvent on the nucleation rates were studied in detail and the affecting mechanism of three key factors was proposed. Furthermore, computational chemistry was also adopted to form a self-consistent interpretation, which links solute–solvent interactions and molecular conformation to nucleation behaviours and crystallization products. More importantly, the formation of solvates was also taken into account in the study of the relationship between solution chemistry and nucleation kinetics.

## 2. Experimental   

### 2.1. Materials   

*p*-Nitro­benzoic acid (PNBA) was purchased from Aladdin Chemistry Co. Ltd, China, and its mass fraction purity was higher than 99%. All selected solvents (chloro­form, aceto­nitrile, methanol, DMSO, DMF, NMP, DMA) were analytical reagent grade with molar purity higher than 99.5% and were obtained from Tianjin Kewei Chemical Technology Co. Ltd, China. Chloro­form-*d* (99.8% D), aceto­nitrile-d_3_ (99.8% D), methanol-*d*_4_ (99.8% D, anhydrous), DMSO-d_6_ (99.5% D) and DMF-d_7_ (99.5% D) were purchased from Aladdin Reagent Co. Ltd of China. All chemicals were used without any further purification.

### 2.2. Single crystal growth and determination   

The single crystals of PNBA form (I), (I)·DMSO solvate, (I)·DMF-solvate, (I)·NMP-solvate and (I)·DMA-solvate were cultivated using the slow solvent evaporation method. A saturated solution (2 ml) of PNBA sealed with plastic film was placed into an oven and kept at 298.15 K. Then, crystals of PNBA form (I) and solvates with suitable sizes for single crystal X-ray diffraction were collected after several days. The single-crystal data collection was conducted at 113 K on a Rigaku Rapid II diffractometer, Mo *K*α radiation (λ = 0.71073 Å). Data collection and processing were performed with *Rapid-auto* (Rigaku/MSC, 2004 ▸). Data reduction and cell refinement were performed with *SHELXS*-97 (Sheldrick, 2008 ▸) and *SHELXL*-97 (Sheldrick, 2015 ▸), respectively.

### 2.3. Computation methods   

Density functional theory (DFT) calculations were performed using the *Gaussian09* program to investigate interactions in (1:1) molecular complexes of PNBA in the seven solvents (Frisch *et al.*, 2009 ▸). The equilibrium geometries of PNBA monomer, PNBA dimer, and 1:1 PNBA-solvent complexes were envisaged based on the single-crystal structure of PNBA form (I) and solvates. Then, these geometries were optimized by hybrid M06-2x function and 6-31+G(d,p) basis set with the Grimme D3 dispersion correction using SMD implicit solvation model (Grimme *et al.*, 2010 ▸; Pratt *et al.*, 2007 ▸). The Grimme dispersion correction allows a good description of weak interactions, such as van der Waals interactions. The binding energy (Δ*E*_bind_) between two molecules is calculated using the following equation:
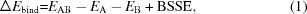
where *E*_AB_ is the energy of the PNBA-solvent complex; *E*_A_ and *E*_B_ are the energies of the isolated monomers PNBA and solvent, respectively. All the energies have been corrected for the zero-point vibrational energies. BSSE is the basis set superposition error and calculated to correct the overestimation of binding energies due to the overlapping of basis functions (Boys & Bernardi, 1970 ▸).

The solvation free energy was calculated by *Materials Studio* (version 7.0; Accelrys Software, 2013 ▸). The amorphous cell model composed of PNBA and solvent was chosen in the study, and each cubic periodic cell contained 1000 molecules. The Geometry Optimization simulation, MD simulation and Solvation Free Energy calculation were employed by Forcite module with *COMPASS* (Condensed-phase Optimized Molecular Potentials for Atomistic Simulation Studies) force field (Bunte & Sun, 2000 ▸; Vyalov *et al.*, 2017 ▸). Other parameters are given in Supporting Information.

### 2.4. Spectroscopy analysis   

^1^H-NMR spectra were measured in chloro­form because the solubility of PNBA in chloro­form is too low to meet the concentration standard for ^13^C-NMR measurement. ^13^C-NMR spectra were measured in aceto­nitrile, methanol, DMSO and DMF, in which the solvents were representative. All ^1^H-NMR and ^13^C-NMR spectra experiments were conducted on a 500 MHz liquid nuclear magnetic resonance spectrometer (Varian Inova 500 MHz) at 298 K after 32 and 256 scans, respectively. Data were processed and analyzed using MestReNova software. ^1^H and ^13^C chemical shifts were determined relative to an internal reference TMS. NMR chemical shifts calculations were performed using Gauge-Independent Atomic Orbital (GIAO) method as implemented in *Gaussian09* (Cheeseman *et al.*, 1996 ▸; Frisch *et al.*, 2009 ▸). All the optimized monomer, dimer or complex structures were calculated using SMD implicit solvation model at the same M06-2x/6-31+G(d,p) level of theory (Pratt *et al.*, 2007 ▸). The reported chemical shifts were relative to those of tetra­methyl­silane (TMS) calculated in the same way.

FTIR spectra results were recorded on an ATR-FTIR spectrometer (ReactIRTM45, Mettler-Toledo) equipped with Duradisc Dicomp probe for solution samples. For each sample, 32 scans were collected over spectra range from 800 to 4000 cm^−1^ at 2 cm^−1^ resolution to investigate the molecular structure of PNBA at different concentrations in the seven solvents tested.

### 2.5. Nucleation kinetics study   

The spontaneous nucleation experiments of PNBA were performed in a round-bottomed jacketed glass batch crystallizer (150 ml). The induction time, which is generally defined as the time period between the moments of establishment of constant supersaturation and formation of detectable crystal particles, was measured for PNBA using a turbidimeter (Crystal Eyes, DMS-2, HEL Ltd) with five to seven compositions in different solvents under 298.15 K. The experimental apparatus is shown in Fig. S1. The experimental procedure is briefly described as follows: a certain amount of PNBA was added into the crystallizer together with different solvents and agitated with a mechanical stirrer at agitation speed of 300 rpm. The temperature was controlled by two thermostats (Julabo CF41, Germany) connected with two t-branch pipes. The temperature accuracy was ± 0.01 K. The temperature was first set at 308.15 K for 1 h to clarify the solution. Then, the channels of t-branch pipes were changed to decrease the temperature to 298.15 K. The moment when system temperature dropped to 298.15 K was used as the starting point of induction time and the end point was the moment when the turbidimeter indicated a sudden increase. Six reproducible experiments were performed at each composition to reduce the experimental error. The relative average deviation (RAD) was calculated by equation (2) to evaluate the accuracy of the data.
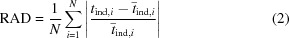
where *N* is the number of experimental measurements under the same condition; *t*_ind,*i*_ and 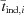 refer to the experimental induction time of experiment *i* and the average value of reproducible experiments, respectively.

According to classical nucleation theory, the dependence of nucleation rate on supersaturation can be described by equations (3) and (4):[Disp-formula fd4]

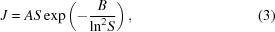


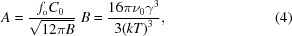
where *J* is the nucleation rate (m^−3^ s^−1^), *A* is the nucleation kinetic parameter; *S* is the degree of supersaturation, *B* is the nucleation thermodynamic parameter, *f*_o_ is the attachment frequency of building units to a nucleus, *C*_0_ is the concentration of nucleation sites, ν_0_ is the molecular volume (m^3^), γ is the interfacial tension (J m^−2^), *k* is Boltzmann constant and *T* is the absolute temperature (K). By plotting the linear function of ln(*J*/*S*) versus 1/ln^2^ *S*, the pre-exponential kinetic factor *A* can be derived from the intercept and the thermodynamic parameter *B* can be obtained from the slope.

## 3. Results and discussion   

### 3.1. Crystallization outcomes and crystal structure data   

The solid forms of PNBA in chloro­form, aceto­nitrile, methanol, DMSO, DMF, NMP and DMA at different temperatures and supersaturations were studied and the results are given in Table 1 ▸. In chloro­form, aceto­nitrile and methanol, the crystallization product is unsolvated polymorphic form (I). From single crystal XRD data, form (I) (CSD refcode NBZOAC 15) belongs to the monoclinic crystal system with the carb­oxy­lic acid 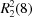 dimer, which is stacked through π–π and —CH⋯π interactions. However, in DMSO, DMF, NMP and DMA, the experiment results showed that PNBA crystallized as the corresponding solvates regardless of supersaturation or temperature. Although pure polymorphic form (I) is more thermodynamically stable (higher melting point) than solvates under atmospheric conditions, form (I) could transform into corresponding solvates in DMSO, DMF, NMP and DMA. Take DMSO for example, form (I) will transform to DMSO-solvate by solvent-mediated transformation in DMSO. The obtained single crystal data showed that DMSO-solvate, DMF-solvate and DMA-solvate were the same crystal forms with their CSD refcodes reported in literature as XIYGIY, XIYFUJ and XIYJEX, respectively (Dash *et al.*, 2019 ▸). However, the single-crystal data of NMP-solvate was first solved and analyzed in this work. The details are listed in Table 2 ▸. Among them, DMSO-solvate belongs to monoclinic crystal system and the space group is 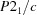, while DMF-solvate, NMP-solvate and DMA-solvate all belong to triclinic crystal system with space group of 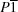. The asymmetric unit of DMSO-solvate consists of two PNBA molecules and two DMSO molecules, while for DMF/NMP/DMA-solvate, the asymmetric unit consists of one PNBA molecule and one solvent molecule, forming a PNBA–solvent heterodimer which was further stacked by face-to-face and/or face-to-side aromatic ring interactions.

According to Price *et al.* (2006 ▸), there are generally two main structural driving forces for the formation of solvates. (1) There are many voids in the packing arrangements of the main molecules, and the entrance of solvent can reduce the voids to improve the packing efficiency (Vippagunta *et al.*, 2001 ▸). (2) The introduction of exotic solvents can form stronger and more stable intermolecular interactions than those involving only the solutes (Tessler & Goldberg, 2006 ▸). According to Table 2 ▸, DMSO-solvate, DMF-solvate, NMP-solvate and DMA-solvate all belong to the second case which can be explained by the carboxyl groups in PNBA which act as donors to form hydrogen bonds with solvents. Besides, the stronger the hydrogen-bond receptor capacity of the solvent is, the higher the solvation strength between the solute and the solvent will be, thus facilitating the self-assembly of solute and solvent molecules to form corresponding solvates. The calculated solute–solvent interactions in Section 3.2 also support this conclusion.

### 3.2. Molecular interactions   

**Figure 1:**
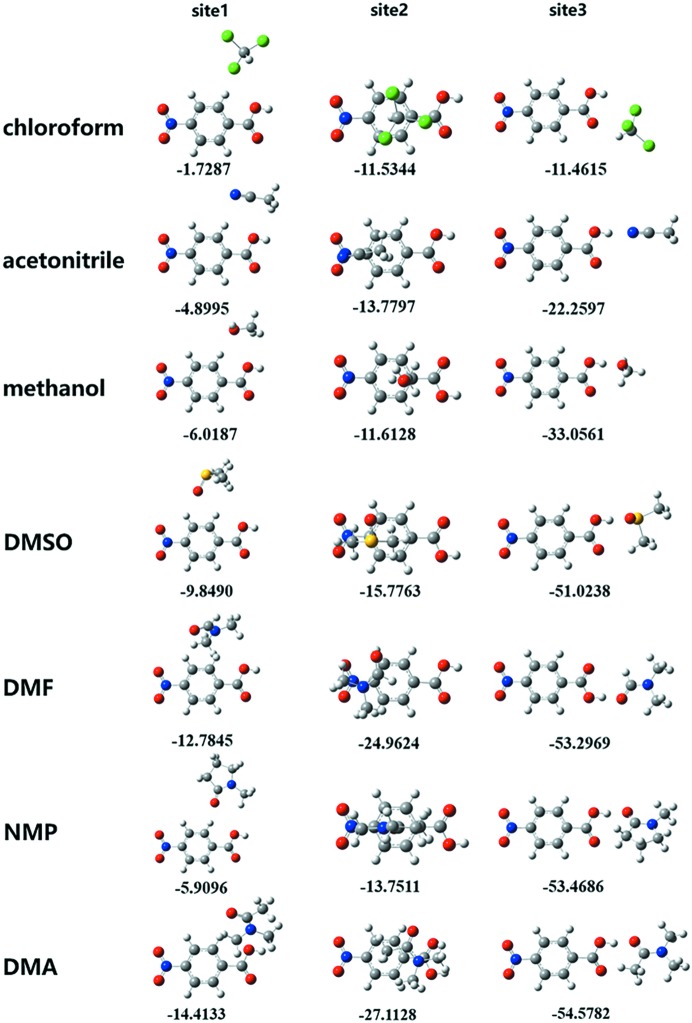
Optimized geometries and binding energies (kJ mol^−1^) for 1:1 PNBA-solvent complexes, calculated at M062X/6-31G(d,p) level. Carbon-grey, hydrogen-white, oxygen-red, nitro­gen-blue, sulfur-yellow, chlorine-green.

It is generally believed that electrostatic potential can be used to predict and explain the relative molecular orientation and the strength of the combination if a complex is mainly assembled by static electricity (such as hydrogen bond, di­hydrogen bond, halogen bond, *etc*). And the more negative (or positive) the electrostatic potential is, the more electrophilic (nucleophilic) the atom is likely to be. Thus, the distribution of van der Waals surface electrostatic potential of molecules can be analyzed and used to predict the most active sites. According to the van der Waals surface electrostatic potential distribution diagram plotted by *Multiwfn* and *VMD* (Fig. S2) (Lu & Chen, 2012*a* ▸,*b* ▸), three sites in PNBA were selected to optimize the 1:1 solute–solvent complexes and calculate the binding energy: benzene ring hydrogens (site 1), benzene ring π-electrons (site 2), and carboxyl hydrogen (site 3). The optimized geometries and binding energy results are shown in Fig. 1 ▸.

It can be seen from Fig. 1 ▸ that almost all the solute–solvent binding energies appear to be the weakest at site 1 and the strongest at site 3. However, the binding energy of site 2 was a bit higher than that of site 3 in chloro­form, and the binding energy of PNBA-chloro­form is the weakest among all solvents for all the three sites. This is because aceto­nitrile, methanol, DMSO, DMF, NMP and DMA all have strong hydrogen-bond acceptors, favouring the formation of heterodimers which can significantly affect the binding energy. Besides, it can also be seen from Fig. 1 ▸ that the strongest solute–solvent binding at site 3 is observed for DMA, followed by NMP, DMF , DMSO, methanol, aceto­nitrile and chloro­form. The four with the strongest binding (DMA, NMP, DMF and DMSO) also lead to the formation of corresponding solvates.

To obtain further insight into the interactions between PNBA and solvents, the total solvation free energies including short-range van der Waals forces and long-range electrostatic interactions were calculated. The calculated results are listed in Table 3 ▸ and the final configurations of simulation are shown in Fig. S3. DMA solvation has the strongest solute–solvent interaction, indicating the highest cohesive strength. Then, a trend of decrease in Δ*G*_solv_ can be seen from NMP, DMF, DMSO, methanol to aceto­nitrile. The weakest calculated strength was from chloro­form, which is consistent with the weakest binding observed for the 1:1 PNBA-chloro­form complex.

### 3.3. Mechanism of molecular self-assembly   

NMR spectroscopy was used to further investigate the molecular interactions of PNBA in various solutions. The NMR chemical shifts can reflect not only the ensemble average interaction of the solution, but also the small changes in the local chemical environment of the molecule. The NMR chemical shifts of PNBA saturated solutions in chloro­form, aceto­nitrile, methanol, DMSO and DMF were measured and the results are listed in Table 4 ▸. It was found that the NMR chemical shifts of low-concentration solutions were slightly different from those of high-concentration solutions in a certain solvent. However, it showed considerable difference (up to several ppms), especially for H_16_ and C_7_, in different solvents, revealing different solute–solvent interactions and different PNBA states.

In order to predict the form of PNBA existing in various solvents, the chemical shifts of PNBA monomer, dimer and solvated form in each solution were simulated by *Guassian09* with SMD implicit solvation model (Frisch *et al.*, 2009 ▸). All of the conformations were optimized or obtained from the corresponding single-crystal structures (Fig. S4). The calculated NMR chemical shifts for each conformation were linearly fitted to experimental values by scaling method. The equation is shown as follows:
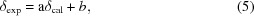
where δ_exp_ is the chemical shift measured by experiments; δ_cal_ is the chemical shift calculated by *Gaussian09* (Frisch *et al.*, 2009 ▸).

The obtained values of *a*, *b* and the fitness *R^2^* are listed in Table 4 ▸. It is evident that the chemical shifts of carboxyl hydrogen H_16_ and carboxyl carbon C_7_ are more sensitive to the molecular conformation and the solvent medium. In chloro­form, the fits between calculated and experimental values were excellent for dimers and unsatisfied for monomers and solvated form. Besides, the calculated chemical shift of H_16_ showed little difference from those of other protons in the chloro­form-solvated form due to the low polarity of chloro­form. The H_16_ chemical shift showed a considerable increase in dimer, indicating a significant deshielding effect, which is consistent with the experimental liquid NMR data. It can be concluded from these results that dimers were preferred over monomers and solvated forms in chloro­form solution. However, the situation was different in aceto­nitrile, methanol, DMSO and DMF. In these solvents, the ^13^C chemical shift fitted better for solvated form than monomers or dimers. The calculated C_7_ chemical shifts of dimers and solvated form in these solvents showed different degrees of increase compared to that of monomers. These differences were caused by the different intermolecular interactions of PNBA-solvent complexes and the different solvents polarity. The deshielding effect of solvated form could describe the experimental behaviours of liquid NMR.

**Figure 2:**
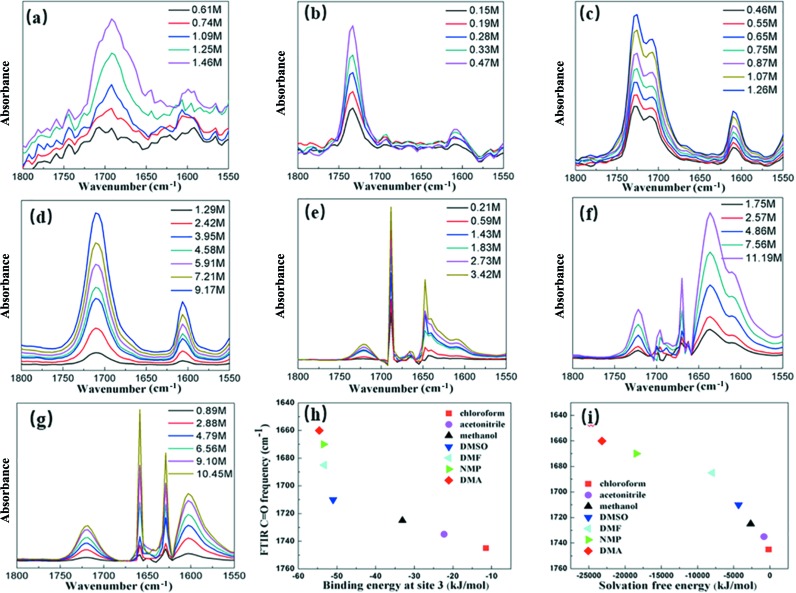
(*a*)–(*g*) FTIR spectra of PNBA solutions in chloro­form, aceto­nitrile, methanol, DMSO, DMF, NMP and DMA at different concentrations; (*h*) relationship between carbonyl stretching and binding energy at site 3; (*i*) relationship between carbonyl stretching and solation free energy.

In order to further confirm the form of PNBA existing in the selected solutions, FTIR spectroscopy was used to monitor the bands of C=O stretching of PNBA in chloro­form, aceto­nitrile, methanol, DMSO, DMF, NMP and DMA. The C=O stretch peak is sensitive to carb­oxy­lic acid dimerization due to the hydrogen bond and the transition dipole coupling between two C=O bonds in the dimer (Dybal *et al.*, 1987 ▸). In general, the frequency of C=O stretch of dimer is 40∼50 cm^−1^ lower than that of monomer. The relative intensity of C=O stretch is also strongly dependent on solution concentration: monomer peak dominates at low concentration while dimer peak dominates at high concentration. Thus, the C=O stretch peaks of monomer and dimer will be visible in the mixture of monomers and dimers. In the FTIR spectra of PNBA in chloro­form [Fig. 2 ▸(*a*)], two C=O stretch peaks at 1745 cm^−1^ and 1695 cm^−1^ can be observed and the frequency splitting is 50 cm^−1^, which agrees with the usual difference of monomer and dimer in C=O stretch frequencies (Fujii *et al.*, 1988 ▸). Furthermore, it can be clearly seen that the relative intensities between the dimer band at 1695 cm^−1^ and the monomer band at 1745 cm^−1^ increased gradually as the solution concentration increased from 0.61 *M* to 1.46 *M*, suggesting a tendency to form carb­oxy­lic acid dimers when increasing PNBA concentration in chloro­form.

As for aceto­nitrile [Fig. 2 ▸(*b*)], there were two C=O stretch peaks at 1735 cm^−1^ and 1695 cm^−1^. The carbonyl groups would not be solvated strongly in this case due to the absence of a hydrogen-bond donor in aceto­nitrile. Thus, due to weakly solvated carbonyl groups, the 1735 cm^−1^ C=O peak was only 10 cm^−1^ red shifted compared to that of monomer. The weak peak at 1695 cm^−1^ suggested the presence of a small amount of dimers in solution. Thus, the formation of dimers may be hindered by the solvation of carboxyl groups in aceto­nitrile. The intensity of 1735 cm^−1^ peak increased more significantly than the intensity of the 1695 cm^−1^ peak when concentration increased. The results further support the conclusion that solvated PNBA was favoured even in aceto­nitrile solution of high concentration.

However, PNBA showed different behaviours in methanol. As shown in Fig. 2 ▸(*c*), there were both a strong band at 1725 cm^−1^ and a weak shoulder band at 1705 cm^−1^ due to C=O stretch. The frequency splitting was only 20 cm^−1^, much smaller than the typical value (50 cm^−1^) of carb­oxy­lic acid monomer-dimer equilibria. Besides, it can also be seen from Fig. 2 ▸(*c*) that the intensity ratio of the two peaks was completely independent of concentration. Thus, the two C=O stretch peaks in methanol can not be derived from PNBA dimerization, even in supersaturated solution. It is most likely that the stronger peak at 1725 cm^−1^ was related to the solvated carbonyl group in methanol (formation of C=O⋯H—O hydrogen bond), and the weaker shoulder peak at 1705 cm^−1^ was the result of the weakly self-associated but not dimerized PNBA molecules. Similar phenomena have been observed for both *N*-methyl­acetamide in methanol and tolfenamic acid in ethanol, with exactly the same splitting frequency (20 cm^−1^) as PNBA in methanol (Du *et al.*, 2015 ▸; Woutersen *et al.*, 2001 ▸).

DMSO, DMF, NMP and DMA have strong hydrogen-bond acceptors and donors, and they can interact with both carboxyl groups and hydrogens of PNBA. The C=O stretch peak of PNBA in DMSO solution was at 1710 cm^−1^ which was 35 cm^−1^ red shifted compared with that of monomer, and the S=O stretch peak of DMSO at 1605 cm^−1^ was 20 cm^−1^ red shifted in comparison with that of pure DMSO solvent (1625 cm^−1^). These results indicated the formation of hydrogen bond in PNBA-DMSO solvate. Similar results were observed in DMF, NMP and DMA, in which the C=O stretch peaks were at 1685 cm^−1^, 1670 cm^−1^ and 1660 cm^−1^ respectively, indicating the formation of hydrogen bonds in corresponding solvates. Figs. 2 ▸(*h*) and 2 ▸(*i*) show the carbonyl stretch versus the binding energy at site 3 and the solvation free energy, respectively. Based on the results, a conclusion can be drawn that stronger solute–solvent interaction will result in stronger deshielding effect of C=O stretch peak.

**Figure 3:**
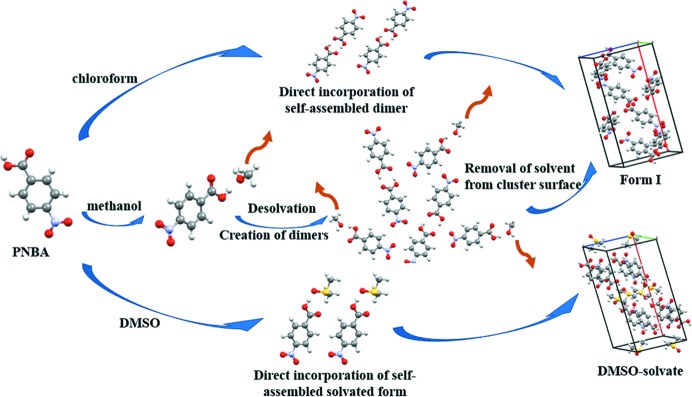
The proposed schematic diagram of molecular self-assembly mechanism during nucleation.

The above conclusions from spectroscopy results indicated distinct solute–solvent interactions and self-assemblies of PNBA in the seven solvents. In chloro­form, the computed results of dimers fitted best with that of NMR and two C=O stretch peaks turned out to be concentration-dependent in FTIR analysis. These results jointly indicated that PNBA would form 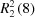 carb­oxy­lic acid dimers in chloro­form. However, PNBA showed different behaviours in aceto­nitrile, methanol, DMSO, DMF, NMP and DMA. C=O stretch peaks had significant red shift, demonstrating that solute–solvent interactions would result in the formation of PNBA-solvent hydrogen-bonded complexes. Form (I) of PNBA was crystallized from chloro­form, aceto­nitrile and methanol, while solvates of PNBA were obtained from corresponding solvents, including DMSO, DMF, NMP and DMA. The results could be explained by the following reasons: Firstly, the similarity between solution chemistry and crystal structure was significant when PNBA crystallized in chloro­form, DMSO, DMF, NMP and DMA. Corresponding PNBA–PNBA homologous dimers or PNBA-solvent heterologous dimers are formed in solution and preserved in the final crystalline structures. However, when PNBA crystallized from aceto­nitrile and methanol, there was no direct correlation between solution chemistry and crystal structure. Although PNBA formed hydrogen bonds with the solvent molecules in solution, the final crystal structures did not contain solvent, ending up with PNBA–PNBA homologous dimers. The structural difference between solution chemistry and crystal synthesizer implies a unique nucleation pathway: the nucleation process contains an additional desolvation process prior to the formation of dimers (Fig. 3 ▸). The solvated PNBA aggregates undergo supramolecular recombination to remove the solvent and gradually form the dense crystal core containing only PNBA–PNBA structures. Firstly, the clusters are likely to contain PNBA-solvent hydrogen-bonded complexes at the initial stage of nucleation. Then, they will be transformed into PNBA–PNBA clusters through dissolvation. Finally, the stable crystal nuclei are eventually formed and they will continue to grow into bigger crystals.

### 3.4. Mechanism of the nucleation process   

The induction time of PNBA in various solvents at different supersaturation was measured and the results are listed in Table S1. All the calculated RADs of reproducible experiments were less than 5%, demonstrating a good reproducibility. The error was mainly caused by the inevitable time lag of the measurement technique. Thus, the fluctuation of measured induction time did not show the random phenomenon of induction time, which has commonly been observed in solutions of small volumes. The measured induction time therefore can be directly applied to estimate the nucleation rates and nucleation kinetic parameters of crystals (Kaschiev, 2000 ▸).

**Figure 4:**
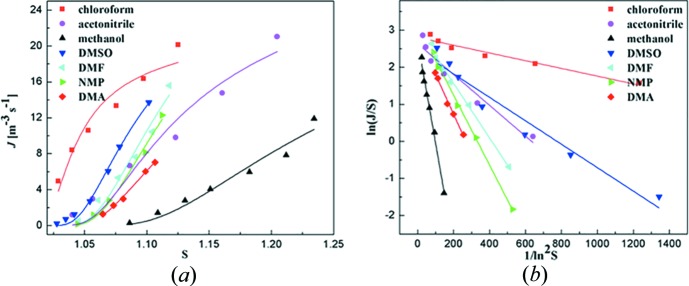
Relationship between (*a*) nucleation rate *J* and supersaturation *S*; (*b*) ln(*J*/*S*) and 1/(ln^2^ *S*).

The crystal nucleation rates *J* were plotted against solution supersaturation *S* in Fig. 4 ▸(*a*). It revealed that, within the experimental supersaturation range, PNBA nucleation appeared relatively favourable in chloro­form and became increasingly difficult in the order of DMSO, DMF, NMP, aceto­nitrile, DMA, and finally methanol. As shown in Fig. 4 ▸(*b*), 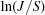 and 1/ln^2^ *S* showed a good linear relationship in all seven solvents. The fitted parameters *A*, *B*, the calculated value of 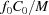 (proportional to the attachment frequency) and interfacial energy γ are listed in Table 5 ▸. It can be seen that the molecular attachment frequency followed the order of DMSO < DMA < NMP < DMF < methanol < aceto­nitrile < chloro­form, which had no obvious relationship with that of nucleation rates data. In contrast, the order of interfacial energy γ was chloro­form < DMSO < DMF < NMP < DMA < aceto­nitrile < methanol, almost the same with that of nucleation rates data. The exception of aceto­nitrile may be due to the effects of solvent’s shape and volume on the nucleation of solvates. Thus, it can be inferred that the interfacial energy, which was closely related to the interactions between solute molecules and solvent molecules, played a crucial role in the nucleation rates of PNBA in these solvents.

**Figure 5:**
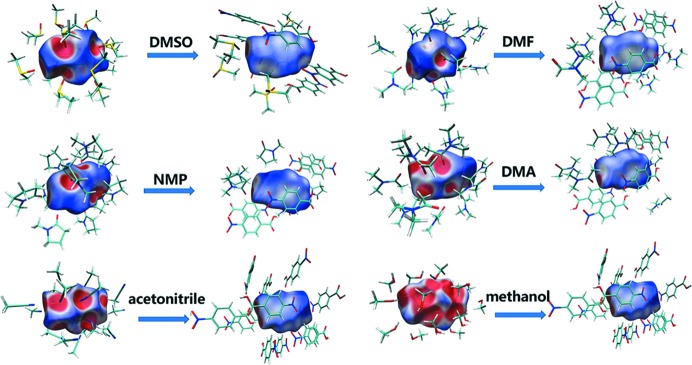
The environment of PNBA molecules and the interaction with surrounding molecules in various solvents and single crystals.

Previous studies have revealed the relationship between nucleation rates and solute–solvent interactions. From the literature, three rules can be concluded as follows: (1) nucleation rates will increase when the conformational structure in solution is similar to the crystalline product (Petit *et al.*, 1994 ▸); (2) the influences of solvents on nucleation may mainly come from interactions between specific sites rather than the overall solvation energy (Khamar *et al.*, 2014 ▸); (3) nucleation rates will decrease when the binding between solvent and solute molecules become stronger (Zeglinski *et al.*, 2018 ▸). Most results obtained in this work can be explained by these three rules. However, some results obtained in this work can not be completely interpreted by these rules since solvates, which are crystal-like but also strongly solvated, were taken into account in this work. As discussed above, the structures of PNBA in solutions of chloro­form, DMSO, DMF, NMP and DMA were similar with those of PNBA in crystalline solids. However, different results were observed when additional dissolvation process were involved, such as in aceto­nitrile and methanol. According to the first rule, the nucleation rates of PNBA in chloro­form, DMSO, DMF, NMP, DMA should be larger than those in aceto­nitrile and methanol. However, experimentally, PNBA nucleated more easily in aceto­nitrile than in DMA. Besides, both the strength of specific site interactions (site 3) and the overall solvation free-energy followed the trend of chloro­form < aceto­nitrile < methanol < DMSO < DMF < NMP < DMA. Whereas, the order of nucleation rates was methanol < DMA < aceto­nitrile < NMP < DMF < DMSO < chloro­form. In DMSO, DMF, NMP and DMA systems, PNBA formed corresponding hydrogen-bonded solvates which did not require complete desolvation, and the specific site structure (site 3) with the strongest interaction was retained in the final crystal structure. For these solvents, higher solvation free energy would result in slower nucleation rates: DMA < NMP < DMF < DMSO. The reason is that PNBA nucleation in these solvents need to overcome the energy required for desolvation at other sites, and the growth of crystalline clusters to the critical nuclei also went through the desolvation process. In order to better understand the nucleation process, the environment of PNBA molecules and their interactions with surrounding molecules in these solvents were visualized by *Multiwfn* and *VMD* (Lu & Chen, 2012*a* ▸; Lu & Chen, 2012*b* ▸), as shown in Fig. 5 ▸. The red areas with the largest electron density represent the strongest interaction, such as hydrogen bond, while the blue areas with the least electron density do not have apparent interaction and the white areas with medium electron density indicate relatively weak interaction, such as π–π stacking in these systems. It can be seen from Fig. 5 ▸ that, in the solution environment, in addition to the red areas at the carboxyl sites of PNBA, there were also relatively strong interactions at other sites which transformed to relatively weak interactions when PNBA crystallized. Thus, apart from the interactions at carboxyl sites, PNBA molecules also had to overcome these interaction forces when they crystallized from liquid phase to solid phase. In other words, the solvation free energy can affect nucleation to some extent.

For solvents which did not form solvates, such as chloro­form, aceto­nitrile and methanol, stronger binding between solvents and PNBA molecules in solution would result in more stable solute–solvent clusters and thus more energy would be required for desolvation process, slowing down the nucleation process. Due to the closest conformational structures in solution to the crystal structures in solid state, the nucleation in chloro­form was the fastest since no desolvation process at carboxyl sites was needed. In addition, the smallest solvation free energy in chloro­form indicated the relatively weak solute–solvent interactions at other sites. Thus, the aggregation of PNBA molecules in chloro­form need to overcome weak interactions, which would also lead to faster nucleation rate, although the dimerization of PNBA in chloro­form was also an effect of the weak solute–solvent interaction. For aceto­nitrile and methanol, the situation was also shown in Fig. 5 ▸. The solvated form at carboxyl sites in solution transformed to the carb­oxy­lic acid 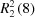 dimers in the final crystal structures, and the stronger interactions (red areas) at other sites transformed to the weaker interactions (white areas). Thus, PNBA molecules had to overcome the overall solvation effects in aceto­nitrile and methanol, which leaded to the relatively low nucleation rates.

However, when all the solvent systems are considered together, no consensus relationship between the conformational structure similarity, the specific site interaction, the solvation free energy and the nucleation difficulty could be summarized. No single factor could individually describe the actual order of the nucleation difficulty and each factor does play a crucial role in certain situation. Therefore, it should be suggested that, none of the three factors: the similarity of the solute in liquid and solid states, the specific site interaction and the overall solvation free energy, could be neglected or underestimated. In fact, they jointly affect the crystal nucleation process.

## 4. Conclusions   

In this work, investigations on the relationship between solution chemistry and nucleation kinetics were carried out by using *p*-nitro­benzoic acid (PNBA) as model compound. It was found that form (I) of PNBA could be obtained in chloro­form, aceto­nitrile and methanol while corresponding solvates would be formed in DMSO, DMF, NMP and DMA. The crystal structures of all these forms were analyzed and discussed. NMR and FTIR spectroscopies were used to analyze the solute species in solution and the results showed that carb­oxy­lic acid dimers of PNBA were thermodynamically favoured in chloro­form, whereas the solvated forms were favoured in aceto­nitrile, methanol, DMSO, DMF, NMP and DMA. The solute species in chloro­form, DMSO, DMF, NMP and DMA were crystal-like while the conformation of solute in aceto­nitrile and methanol was unlike that in the crystal. In aceto­nitrile and methanol, one thermodynamically driven desolvation step was required to form the dense crystal nuclei containing only PNBA molecules.

Computational chemistry based on density functional theory (DFT) was used to calculate the intermolecular interactions and the results revealed that both the solute–solvent interactions at specific sites and the overall solvation free energy followed the order: chloro­form < aceto­nitrile < methanol < DMSO < DMF < NMP < DMA. The nucleation kinetic results of PNBA showed an apparent solvent-dependent behaviour, with the nucleation difficulty in the order of chloro­form < DMSO < DMF < NMP < aceto­nitrile < DMA < methanol. Based on the above results, it can be inferred that the structural similarity between solution chemistry and crystal structure, the interactions between specific sites and the overall solvation strength jointly affect the nucleation process. The results of this work confirm the importance of the pre-assembly and desolvation processes during the crystal nucleation. However, since the nucleation of crystals is complicated and many factors could affect the nucleation process, much more work needs to be carried out to fully understand the nucleation phenomenon.

## Supplementary Material

Crystal structure: contains datablock(s) I.NMP. DOI: 10.1107/S2052520619009478/bm5112sup1.cif

Structure factors: contains datablock(s) I.NMP. DOI: 10.1107/S2052520619009478/bm5112I.NMPsup2.hkl

Supporting information file. DOI: 10.1107/S2052520619009478/bm5112sup3.pdf

CCDC reference: 1906930

## Figures and Tables

**Table 1:** Crystallization products of PNBA in various solvents

	Crystal form
Chloro­form	Form (I)
Aceto­nitrile	Form (I)
Methanol	Form (I)
DMSO	DMSO-solvate
DMF	DMF-solvate
NMP	NMP-solvate
DMA	DMA-solvate

**Table 2:** Selected crystallographic data of form (I), DMSO-solvate, DMF-solvate, NMP-solvate and DMA-solvate of PNBA

	Form (I)	DMSO	DMF	NMP	DMA
Empirical formula	C_7_H_5_NO_4_	C_9_H_11_NO_5_S	C_10_H_12_N_2_O_5_	C_12_H_14_N_2_O_5_	C_11_H_14_N_2_O_5_
Formula weight	167.12	245.25	240.22	266.25	254.24
Crystal system	Monoclinic	Monoclinic	Triclinic	Triclinic	Triclinic
Space group	*C*2/*c*	*P*2_1_/*c*	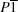	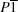	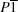
*a* (Å)	21.136 (4)	22.714 (5)	6.2359 (10)	7.1402 (4)	7.2454 (3)
*b* (Å)	5.0489 (10)	7.6639 (15)	7.3281 (2)	7.5689 (4)	7.5178 (3)
*c* (Å)	12.904 (3)	13.026 (3)	12.5359 (3)	11.9955 (5)	11.9625 (4)
α (°)	90	90	102.105 (2)	98.844 (4)	92.682 (3)
β (°)	97.00 (3)	104.68 (3)	99.957 (2)	102.693 (4)	107.121 (3)
γ (°)	90	90	91.211 (2)	100.077 (5)	105.088 (3)
Volume (Å^3^)	1366.9 (5)	2193.5 (9)	550.70 (2)	610.09 (6)	595.85 (4)
Density (g cm^−3^)	1.624	1.485	1.449	1.449	1.417
*Z*	8	8	2	2	2

**Table 3:** Solvation free energy calculated by *Material Studio 7.0*

	Δ*G*_id_ (kJ mol^−1^)	Δ*G*_vdw_ (kJ mol^−1^)	Δ*G*_elec_ (kJ mol^−1^)	Δ*G*_solv_ (kJ mol^−1^)
Chloro­form	50.70	34.19	−264.18	−179.31
Aceto­nitrile	90.00	166.07	−1081.28	−825.23
Methanol	505.65	373.40	−3554.05	−2674.97
DMSO	7311.32	2717.33	−14367.87	−4339.17
DMF	2650.79	849.59	−11470.51	−7970.13
NMP	8057.91	1868.21	−28376.39	−18450.22
DMA	9705.75	−1645.46	−31262.14	−23201.84

**Table 4:** Experimental and calculated chemical shifts for H_16_, H_9_, H_5_, H_11_, H_3_, C_7_, C_6_, C_4_, C_2_ and C_1_ *a* and *b* are the empirical parameters of equation (5); *R* ^2^ is the fitting coefficient. The values of *a*, *b* and *R^2^* are derived from linear regression by scaling method.

	Experimental	Computed mono	Computed dimer	Computed solvated
Chloro­form				
H_16_	11.17	8.63	13.44	8.71
H_9_	8.37	8.55	8.67	8.67
H_5_	8.35	8.46	8.63	8.60
H_11_	8.31	8.32	8.49	8.50
H_3_	8.27	7.73	8.47	8.29
*a*		0.1353	1.7139	0.0718
*b*		7.1344	−5.7037	7.9152
*R* ^2^		0.2304	0.9998	0.2972
Aceto­nitrile				
C_7_	166.08	166.98	173.13	170.72
C_6_	151.71	155.72	157.10	154.49
C_4_	136.52	137.68	137.64	140.03
C_2_	131.79	135.75	135.75	135.60
C_1_	124.57	125.56	127.00	128.85
*a*		0.9954	1.1174	1.0033
*b*		2.8538	−12.695	3.3344
*R^2^*		0.9905	0.9952	0.9982
methanol				
C_7_	166.41	168.51	173.34	173.94
C_6_	150.76	166.16	157.37	154.33
C_4_	136.42	148.15	137.25	140.76
C_2_	130.76	145.96	136.23	135.84
C_1_	123.36	135.86	127.26	129.26
*a*		0.7814	1.0843	1.0307
*b*		42.324	−7.1764	0.9435
*R* ^2^		0.9204	0.9882	0.9935
DMSO				
C_7_	175.30	167.20	173.11	173.04
C_6_	159.53	154.86	157.08	153.95
C_4_	145.85	138.61	137.62	142.08
C_2_	140.20	135.24	135.76	135.26
C_1_	133.25	127.95	127.00	128.48
*a*		0.9483	1.1097	1.0458
*b*		1.7562	−21.26	−11.177
*R^2^*		0.9936	0.9908	0.9965
DMF				
C_7_	166.59	170.97	175.73	170.56
C_6_	151.09	155.64	155.85	153.95
C_4_	137.47	137.29	137.95	142.04
C_2_	131.51	135.79	136.28	135.32
C_1_	124.41	127.82	127.80	128.34
*a*		1.0638	1.1342	0.9909
*b*		−1.938	−14.579	5.1279
*R* ^2^		0.9884	0.9873	0.9987

**Table 5:** Summary of the classical nucleation theory (CNT) kinetic and thermodynamic parameters

	*A* (m^−3^ s^−1^)	*B* (×10^2^)	*f*_0_ *C*_0_/*M* (×10^2^)	γ (mJ m^−2^)
Chloro­form	16.51	0.10	30.02	0.50
Aceto­nitrile	13.97	0.42	2.50	0.79
Methanol	15.20	2.78	1.62	1.48
DMSO	11.63	0.32	0.22	0.52
DMF	20.82	0.74	1.35	0.66
NMP	22.92	0.94	1.06	0.69
DMA	17.64	1.07	0.64	0.71
