# Supplementary material for: Insight into the role of pre-assembly and desolvation in crystal nucleation: a case of p-nitro­benzoic acid
Source: Acta Crystallogr B Struct Sci Cryst Eng Mater. 2019 Sep 18;75(Pt 5):845–54. doi: 10.1107/S2052520619009478 (PMC6816033; doi:10.1107/S2052520619009478)
Supplement: Supplementary file 3 [file b-75-00845-sup3.pdf]

STRUCTURAL SCIENCE
CRYSTAL ENGINEERING
MATERIALS

Volume 75 (2019)

Supporting information for article:

Insight into the role of pre-assembly and desolvation in crystal nucleation: a case of p-nitrobenzoic acid

Shuyi Zong, Jingkang Wang, Hao Wu, Qi Liu, Yunhui Hao, Xin Huang, Dehui Wu, Guanchen Zhou and Hongxun Hao

S1. Solvation free energy calculation

The solvation free energy was calculated by Materials Studio (MS) 7.0. The amorphous cell model composed of PNBA and solvent in terms of molar solubility was chosen in the study, and every cubic periodic cell contained 1000 molecules. The Geometry Optimization simulation, both MD simulation and Solvation Free Energy calculation were calculated by Forcite module with *COMPASS* (Condensed-phase Optimized Molecular Potentials for Atomistic Simulation Studies) force field (Bunte et al., 2000; Vyalov et al., 2017) which was used to describe the interaction throughout the whole simulations at a fully atomistic level, and the temperature was controlled by Nosé-Hoover-Langevin (NHL) thermostat. The Smart method combining conjugate gradient and steepest descent approach is applied to the energy minimization process, which speeds up the computation. First, the periodic cell was subjected to 100,000-step MM-based geometry optimization to remove the irrelevant contacts. Then, the NVT ensemble dynamic simulation was carried out at the experimental temperature to ensure that the system was in a good state of relaxation and balance. The simulation time was set to 1000 ps and the time step of each dynamic process was set to 1 fs. The van der Waals interaction was computed by atom-based cutoff distance of 15.5 tÅ and the electrostatic interaction was calculated by Ewald summation method with accuracy of 0.418 J/mol. The energy deviation was limited to 209,000 kJ/mol. The obtained configuration served as the initial structure for the solvation free energy calculation which was simulated by thermodynamic integration method. The solvation free energy ΔG_{solv} is the sum of the ideal free energy (ΔG_{id}), the van der Waals free energy (ΔG_{vdw}) and the electrostatic free energy (ΔG_{elec}), as follows:

$$\Delta G_{solv} = \Delta G_{id} + \Delta G_{vdw} + \Delta G_{elec} \quad (1)$$

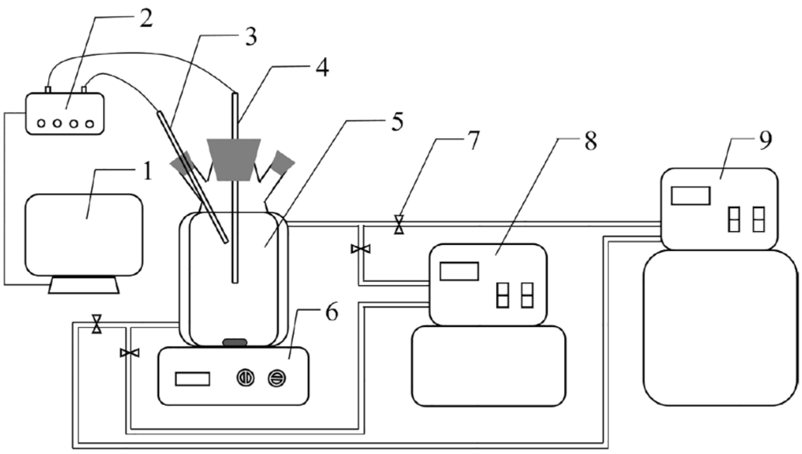

Figure S1 Experimental apparatus for induction time measurements. (1. Data acquisition computer; 2. Turbidimeter controller; 3. Temperature probe; 4. Turbidity sensor; 5. Glass crystallizer with jacket; 6. Magnetic stirrer; 7. Water stop pliers; 8. Thermostat used for controlling high temperature; 9. Thermostat used for controlling low temperature.)

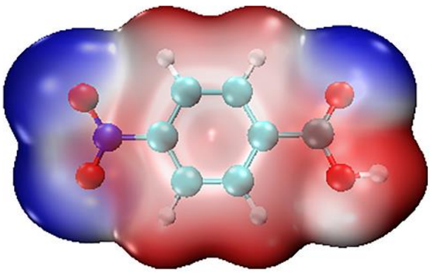

Figure S2 The van der Waals surface electrostatic potential of PNBA plotted by Multiwfn and VMD (Lu & Chen, 2012a; Lu & Chen, 2012b).

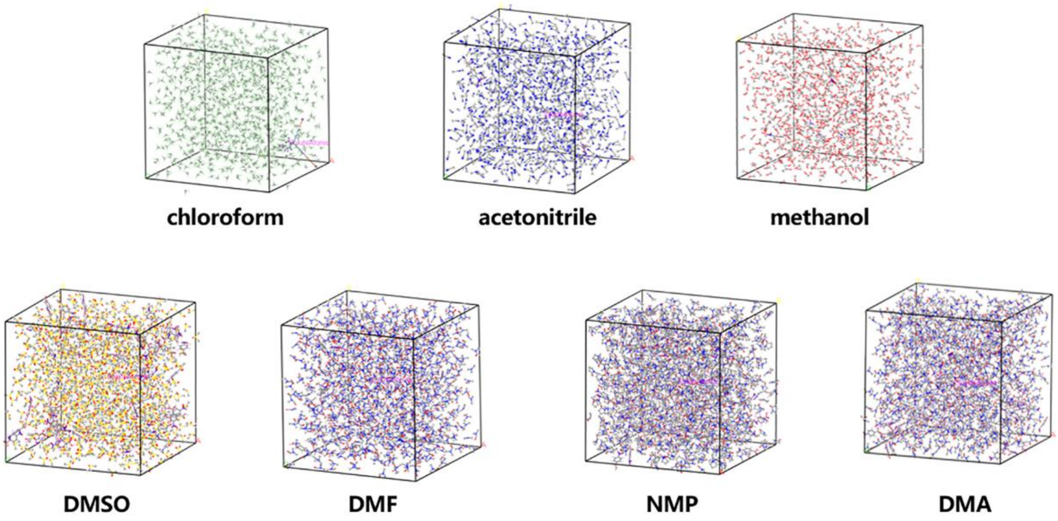

Figure S3 The final configurations of solvation free energy simulation

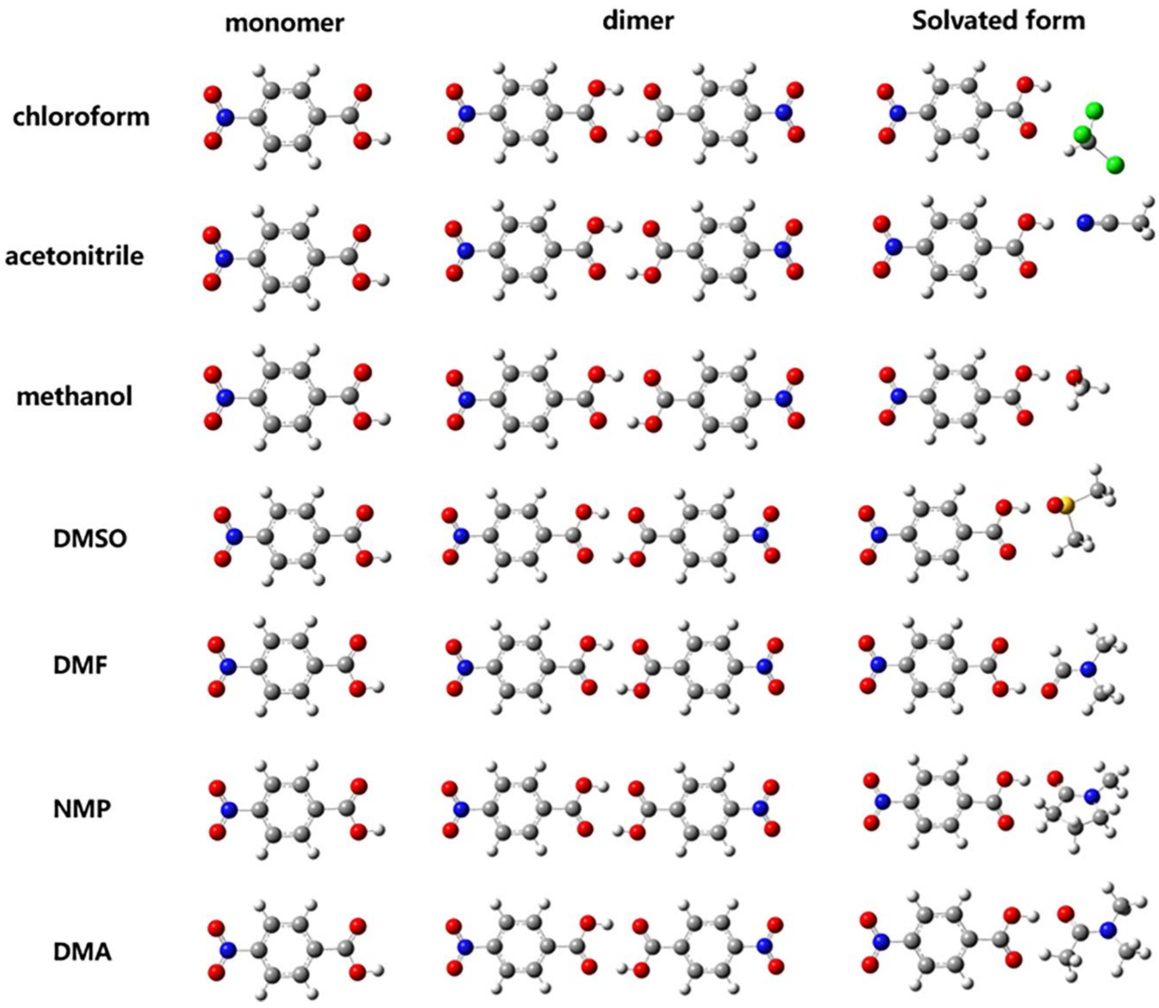

Figure S4 Optimized conformations for NMR calculation (using SMD implicit solvation model)

Table S1 Data of nucleation rates calculated by induction time measurements.

Supersaturation *S*	Average induction time / min	nucleation rates *J* / m^{-3} s^{-1}	$1/\ln^2 S$	$\ln(J/S)$
		chloroform		
1.029	22.51	4.936	1234	1.568
1.040	13.23	8.398	654.6	2.089
1.053	10.50	10.58	375.9	2.308
1.075	8.33	13.34	189.1	2.518
1.097	6.79	16.36	115.8	2.702
1.125	5.52	20.13	71.96	2.884
		acetonitrile		
1.040	93.67	1.186	641.5	0.1312
1.056	37.42	2.969	332.6	1.034
1.086	16.65	6.673	145.9	1.815
1.123	11.33	9.807	74.13	2.167
1.161	7.520	14.78	45.08	2.544
1.205	5.280	21.04	28.85	2.860
		methanol		
1.086	415.4	0.2677	146.2	-1.401
1.109	78.77	1.411	93.85	0.2408
1.131	40.11	2.778	66.32	0.8961
1.151	27.33	4.065	50.55	1.262
1.183	18.67	5.952	35.60	1.616
1.212	14.17	7.843	27.07	1.868
1.234	9.330	11.91	22.58	2.267
		DMSO		
1.028	482.5	0.2303	1343	-1.496
1.035	154.6	0.7186	851.3	-0.3647
1.042	89.24	1.245	596.0	0.1782
1.054	41.21	2.696	358.3	0.9390

1.069	18.33	6.062	225.3	1.735
1.078	12.67	8.770	176.9	2.096
1.102	8.100	13.72	106.8	2.522
		DMF		
1.045	212.00	0.5241	508.6	-0.6904
1.061	39.31	2.827	285.9	0.9799
1.077	20.79	5.345	181.8	1.602
1.091	14.55	7.637	132.1	1.946
1.104	10.62	10.46	101.4	2.249
1.118	7.120	15.61	80.30	2.636
		NMP		
1.045	663.5	0.1675	527.3	-1.831
1.057	94.87	1.171	324.3	0.1025
1.070	39.67	2.801	221.4	0.9627
1.099	13.64	8.146	112.8	2.003
1.112	9.030	12.30	88.17	2.404
		DMA		
1.065	87.03	1.277	254.8	0.1816
1.073	49.68	2.237	199.8	0.7342
1.081	37.29	2.980	164.9	1.014
1.099	18.43	6.029	112.4	1.702
1.107	15.79	7.037	97.53	1.850

References

Bunte, S. W. & Sun, H. (2000). *J Phys Chem B*, **104**, 2477-2489.

Lu, T. & Chen, F. (2012a). *Acta Phys.-Chim. Sin*, **28**, 1-18.

Lu, T. & Chen, F. (2012b). *J. Comput. Chem*, **33**, 580-592.

Vyalov, I., Vaksler, Y., Koverga, V., Miannay, F. A., Kiselev, M. & Idrissi, A. (2017). *J Mol Liq*, **245**, 97-102.
